# Genome-wide gene expression profiling of human mast cells stimulated by IgE or FcεRI-aggregation reveals a complex network of genes involved in inflammatory responses

**DOI:** 10.1186/1471-2164-7-210

**Published:** 2006-08-16

**Authors:** Manikandan Jayapal, Hwee Kee Tay, Renji Reghunathan, Liang Zhi, Kah Kiong Chow, Mary Rauff, Alirio J Melendez

**Affiliations:** 1 Department of Physiology, National University of Singapore, Singapore; 2 Department of Obstetrics & Gynecology, Gleneagles Medical Centre, Singapore; 3 Department of Obstetrics & Gynecology, National University of Singapore, Singapore

## Abstract

**Background:**

Mast cells are well established effectors of IgE-triggered allergic reactions and immune responses to parasitic infections. Recent studies indicate that mast cells may play roles in adaptive and innate immunity, suggesting an innovative view of the regulation of immune responses. Here, we profiled the transcriptome of human mast cells sensitized with IgE alone, or stimulated by FcεRI aggregation.

**Results:**

Our data show that among 8,793 genes examined, 559 genes are differentially regulated in stimulated mast cells when compared with resting/unstimulated mast cells. The major functional categories of upregulated genes include cytokines, chemokines, and other genes involved in innate and adaptive immune-responses. We observed the increased expression of over 63 gene-transcripts following IgE-sensitization alone. Our data was validated using Real-Time-PCR; ELISA and western blot. We confirmed that IgE alone does not trigger mast cell-immediate responses, such as calcium signals, degranulation or protein-phosphorylation.

**Conclusion:**

This report represents a substantial advance in our understanding of the genome wide effects triggered by "passive sensitization" or active stimulation of human mast cells, supporting mast cells' potential involvement in a wide range of inflammatory responses.

## Background

Mast cells are best known for their role in immunoglobulin E (IgE)-dependent allergic responses as one of the most powerful reactions of the immune system [1]. Recent studies suggest that mast cells may also be involved in innate and adaptive immunity by producing high levels of chemokines and cytokines [1-3]. Mast cells are derived from haematopoietic progenitor cells that enter nearly all vascularized tissues, where they complete their maturation and, in some cases, can migrate into epithelia [1-4]. After appropriate activation, such as the aggregation of the high affinity IgE receptor (FcεRI), mast cells can produce a range of pro-inflammatory mediators, including cytokines and chemokines [5,6]. Crosslinking of FcεRI-bound IgE with multivalent antigen initiates the activation of mast cells by promoting the aggregation of FcεRI [7,8]. This FcεRI-dependent cell activation process has three major outcomes: degranulation, secretion of preformed mediators stored in cytoplasmic granules – these granules contain vasoactive amines, neutral proteases, proteoglycans and some cytokines and growth factors; the *de novo *synthesis of pro-inflammatory lipid mediators (such as eicosanoids); and the synthesis and secretion of cytokines and chemokines [4-8].

Mast cells are regarded as key effector cells in IgE-associated immediate hypersensitivity reactions and allergic conditions, as well as in certain immune responses to parasites [9]. Because the IgE dependent release of proinflammatory mediators can begin within minutes of antigen challenge, the crucial role of mast cells in acute allergic reactions, such as anaphylaxis and acute attacks of atopic asthma, is now widely accepted [4,5,7,8]. On the other hand, the role of mast cells in chronic inflammation and other long-term tissue changes that are observed in some IgE associated diseases, including asthma, still debated [10]. However, in recent studies it is suggested that mast cells can markedly enhance antigen-dependent airway hyper reactivity, airway eosinophil infiltration, and the increased number of proliferating cells in the airway epithelium [11-14]. These reports and other lines of evidence, suggest that a key role of mast cells in IgE-associated immune responses is to amplify both acute and long-term local tissue responses to relatively weak biological signals [5,9,11,12]. Moreover, through their ability to release immunoregulatory cytokines, and perhaps through other mechanisms, mast cells might also influence the development, strength and persistence of T-helper 2 cell-associated immunity. However, such immunoregulatory functions of mast cells have not been fully characterized. Mast cells have also been proposed to play a role in mediating bacterial clearance by releasing cytokines, and by ingesting and killing opsonized bacteria, suggesting that mast cells have a physiological role in modulating host defenses against infectious agents [13].

Mast cell products, such as tryptase and histamine, can influence the immune-response by recruiting neutrophils or by activating other immune-effector cells [14]. These previous findings show that mast cells, apart from being principal players in allergies, appear have effects in the initiation and regulation of innate immunity. There is increasing evidence demonstrating that the innate and adaptive immune systems cooperate through shared signaling mechanisms, and very recently it was shown that mast cells may regulate the recruitment of T-cells to lymph nodes [15]. Several groups have carried out limited gene expression profiles of mast cells, without any stimuli [16-18]; stimulated by cytokines [19]; triggered by antigens and in response to the inhibition of calcium-ATPase [20]; or stimulated by TLR4 [21]; or focused on the cytokine/chemokine profile triggered by FcεRI aggregation [21-23]. A recent study has shown that IgE alone can induce the release of the interlukin-8 (IL-8) and of the monocyte chemoattractant protein 1 (MCP-1) [24]. However, many aspects of the genetic responses triggered by IgE-antigen on human mast cells are still unclear, such as the overall genetic responses to IgE sensitization alone, or wide-genome expression profile triggered by FcεRI aggregation. Thus, we decided to analyze the differential gene expression profile of 8,793 fully-annotated genes, after IgE sensitization, and following FcεRI aggregation.

The data we present here show that, over 559 genes were differentially regulated in the stimulated cells, when compared to unstimulated cells (basal). A substantial number of genes were regulated by IgE sensitization alone; and following FcεRI aggregation, a wide range of genes were triggered, including genes for cytokines, chemokines, transcription factors, anti-apoptosis, and several genes involved in innate and acquired immunity. Some of the most prominent findings are the upregulation of proinflammatory cytokines and chemokines, involved in innate and adaptive immunity. We also observed the upregulation of several receptors involved in innate immune reactions. We confirmed that IgE alone does not trigger mast cell-immediate responses, such as calcium signals, degranulation or protein-tyrosine phosphorylation, whereas FcεRI aggregation did indeed trigger these immediate responses. Thus, these results represent a substantial advance in our understanding of the genome-wide effects triggered by the physiological "passive sensitization" or "active stimulation" of human mast cells, and suggest that mast cell activation may not only play a pivotal role in allergic responses, but may influence the regulation of other inflammatory immune responses.

## Results

### Generation of mast cells

**Figure 1:**
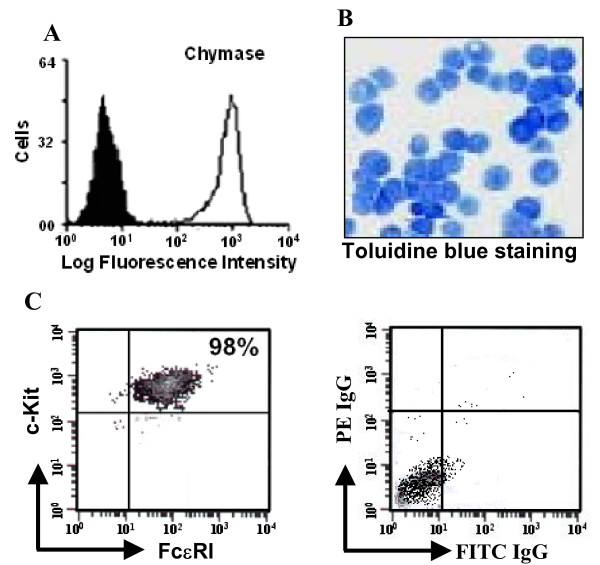
**Purity of Mast cells**. **A. Flow cytometry analysis of intracellular chymase expression**. Human cord blood-derived mast cells labelled for intracellular mast-cell chymase expression (solid line), isotype control (Black area). Results shown are representative of four separate experiments. **B. Toluidine blue staining of mast cells**. Fluorescence microscopy of human cord blood-derived mast cells stained with Toluidine blue. Results shown are representative of at least three separate experiments. **C. Flow cytometry analysis of cell surface expression of c-kit and FcεRI**. Human cord blood-derived mast cells labelled for cell-surface expression of c-kit and FceRI (left panel), and isotype controls (right panel). Results shown are representative of four separate experiments.

Perhaps the most important factor for mast cell development, survival and proliferation is Stem-Cell Factor (SCF or c-KIT ligand) [4], although several lines of evidence also indicate IL-3 as a crucial factor for mast cell development, survival and proliferation [4]. Human mast cells are very difficult to isolate in numbers that would allow for wide-genome expression profiling studies: for that reason we isolated CD34+ progenitor hematopoietic cells from umbilical cord blood and differentiated them to mast cells by culturing with the cytokines SCF and IL-3. After culturing CD34^+ ^progenitor cells with cytokines for 5–6 weeks, cells were characterized as Mast Cells by flow cytometry as positive for the specific mast cell marker intracellular chymase; positive c-Kit/CD117^+ ^and FcεRI^+^, and by Toluidine blue staining (Figure 1). Purity was estimated at >97%.

### Differential gene expression profile in stimulated human mast cells

**Figure 2:**
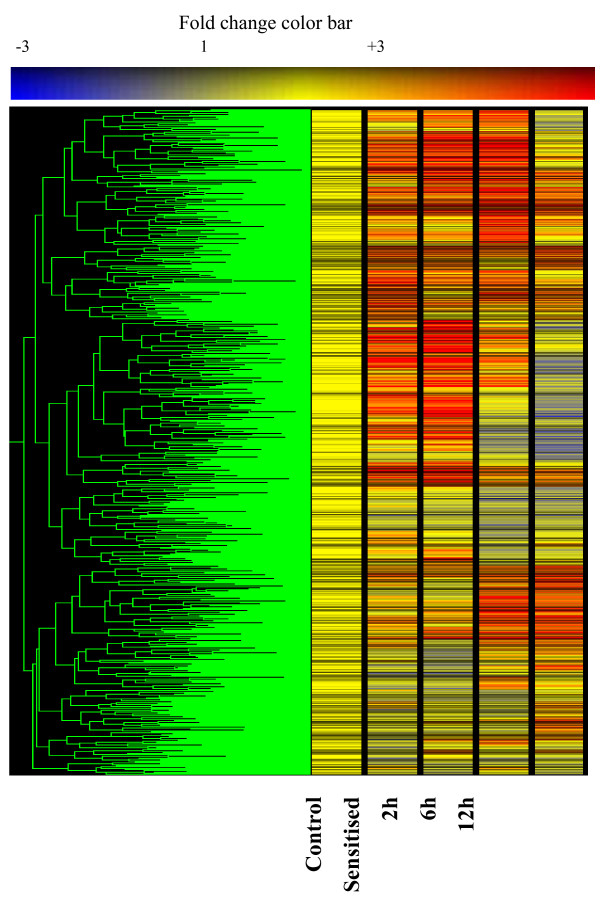
**Changes in gene expression in human mast cells following IgE sensitization and FcεRI aggregation**. Changes in expression over control of human cord blood derived mast cells that were activated by IgE sensitization and FcεRI crosslinking for different time points (2 hr, 6 hr and 12 hr). Labelled cRNA from cell of each time point were hybridized to Human Genome Focus array and signals were scanned after fluidics. The data was analyzed as described in material and methods and analysis revealed differential expression of 760 genes between resting and stimulated mast cells with statistical significance (P ≤ 0.05). Agglomerative average-linkage hierarchical clustering of the five different experimental conditions was obtained for selected groups of genes using GeneSpring 7.0. Each colored box represents the normalized expression level of a given gene in a particular experimental condition and is colored according to the color bar. The data represent average of four independent experiments.

**Figure 3:**
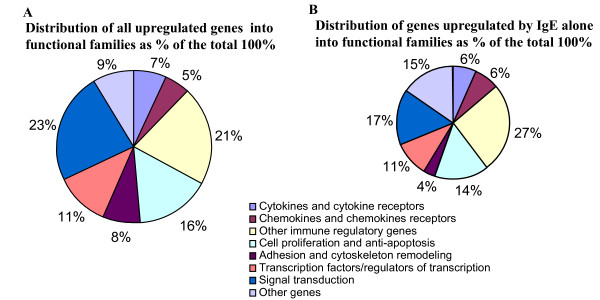
**Pie chart showing the percentage distribution of the upregulated genes**. **A. Percentage distribution of the total amount of genes upregulated**. All the genes, observed to be upregulated at least 2-fold at any given time point, were distributed, according to their biological function described in table 1. **B. Percentage distribution of genes upregulated by IgE alone**. Distribution, according to biological function, of the genes observed to be upregulated during IgE sensitization (IgE alone).

Our aim is to study the global gene expression pattern induced by IgE sensitization and FcεRI aggregation on human mast cells. Gene expression analysis using cDNA or oligo-DNA microarrays has proven to be a sensitive method to develop and refine the molecular determinants of several human disorders, including cancer and autoimmune diseases. We analyzed the expression pattern of 8,793 transcripts from the stimulated mast cells, and compared the expression patterns with control/unstimulated samples. The complete gene expression data of our experiments, representing 8,793 probeID is available at the NCBIs Gene Expression Omnibus [25], and is accessible through GEO Series accession number GSE1933 (25). The microarray analysis revealed that 760 genes (~8.6%) were differentially expressed between resting and stimulated mast cells with statistical significance (P ≤ 0.05), which were hierarchically clustered (Figure 2). Because of the relatively large number of genes that were differentially regulated, we focused on genes that were upregulated by at least a 2-fold in any time point of mast cell stimulation. Of the 760 genes, 58 genes were initially upregulated (at least 2-fold) by IgE-sensitization alone (Table. 1), and a total of 115 genes were overexpressed (by 2-fold or more in at least one time point), during the time course of mast cell activation by IgE-alone or after crosslinking FcεRI (Table. 1). In order to examine the global characteristics of these genes, we used the Gene Ontology Consortium database for Biological processes [26]. **U**sing this database we analyzed the genes that were upregulated by at least 2-fold, thus, allowing us to separate the 115 genes, into the following functional families: (a) cytokines and cytokine receptors; (b) chemokines and chemokine receptors; (c) other immunoregulatory genes; (d) cell proliferation and anti-apoptosis; (e) adhesion and cytoskeleton remodeling; (f) transcription factors and regulators of transcription; (g) signal transduction; (h) genes involved in other cellular functions "others" (Table 1 & Figure 3)

### Upregulation of genes by IgE sensitization

Our study revealed that substantial changes in gene expression in response to monomeric-IgE sensitization alone. In order to ensure that the human IgE (IgE, Cat: 30-AI05, Lot number A01071004, Fitzgerald, Concord, MA), used in this study was indeed monomeric IgE, prior to each experiment; we run a sample of the IgE through non-denaturing polyachrylamide-gel-electrophosys (non-reducing-PAGE). No aggregates were observed at any time (data not shown).

We focused the analysis of our data on genes that were upregulated by at least 2-fold, over basal levels, and identified 58 genes that were increased by IgE sensitization alone (Table. 1). We then separated them into different categories, based on their biological function (determined by public databases). Among the most prominent findings was the upregulation of genes coding for the cytokines IL-1β (3.3 fold), IL-6 (2.7 fold), and CSF1 (1.6 fold); genes coding for the chemokines IL-8 (CXCL8) (2.1 fold), MIP1β (CCL4) (3.5 fold), MCP3 (CCL7) (2.1 fold), GROα (CXCL1) (2.3 fold) and GROγ (CXCL3) (1.6 fold), were also upregulated. Other than these, several genes coding for other receptors involved in immune-responses; immunoregulatory genes; adhesion and/or cytoskeleton remodeling; regulators of apoptosis; signal transduction; transcription factors; were also upregulated by monomeric IgE (Table. 1). Thus, these results suggest that "passive" sensitization of mast cells, with monomeric IgE, may not only prime mast cells to be ready for the challenge to come, but that mast cells may also have the potential to trigger the priming and/or recruitment of other immune-effector cells.

### Gene profile of upregulated genes during various time points of mast cell stimulation cytokines/cytokine receptors and chemokines/chemokine receptors

An interesting finding in our study was upregulation of genes coding for the pyrogenic and proinflammatory cytokines IL-1β, IL-6 (Table. 1a). One of the key roles for IL-1β is to trigger the upregulation of key proinflammatory proteins [27]. We also observed the upregulation of *PTX3 *(pentaxin-related gene) (Table. 1c): PTX3 is a protein involved in inflammation that is rapidly upregulated by IL-1β. Other cytokine genes that were upregulated are *CSF1 *(a growth promoting cytokine), and the cytokine receptors *IL1R1, IL27RA, TNFRSF9*, *TNFRSF12 *and *IL1RN *(Table. 1a). We also found that the level of expression of genes coding for the chemokines IL-8 (CXCL8), CCL7 (MCP3), CCL4 (MIP1β) and CXCL1 (GROα) was increased (Table 1b). IL-8 plays a major role in inflammatory responses mainly due to its ability to recruit and activate neutrophils. CCL7, CCL4 and CXCL1 are known to recruit monocytes, NK, basophils, dendritic cells and TH2 cells. Moreover, our data also show that several other chemokine genes were upregulated during mast cell stimulation: these include CCL5 (RANTES), *CXCL3 *(GROγ), and the chemokine receptor *CCRL2 *(Table. 1b). These data suggest a key role for FcεRI in triggering an a wide range of inflammatory responses, as the over-expression of cytokines and chemokines is a prerequisite to triggering inflammation, including vascular permeability, and leukocyte and lymphocyte recruitment, differentiation and activation.

### Other immune-regulatory genes

Several genes involved in innate and adaptive immune-response were upregulated at least by 2-fold during mast cell stimulation: these include the Toll Like Receptor 2 (*TLR2*), as well as several genes of the TNFα signaling pathways, including *TNFAIP6 *(Table. 1c). These findings support previous reports of the role of mast cells in innate immunity and antibacterial activity [21]. Transcripts of genes LIF, *CD69*, and *CD83 *were also upregulated, as were the major histocompatibility complex genes (*HLA-DQB1*, and *HKE2*) and inhibitory receptor of IgG, *FCGR2B *(Table. 1c). The generation of transcripts for such genes suggests that mast cells can acquire characteristics typical of cells involved in innate and adaptive immune responses.

### Cell proliferation and anti-apoptosis

Several genes involved in cell proliferation and survival, such as *PDGFA *(platelet-derived growth factor alpha polypeptide), *PDGFB *(platelet-derived growth factor beta polypeptide), *PBEF1 *(pre-B-cell colony-enhancing factor), *TIEG *(TGFB inducible early growth response), and *INSIG1 *(insulin induced gene 1) were upregulated, as well as several anti-apoptosis genes including *TNFAIP3 *(TNF-alpha-induced protein 3), *TNFAIP8 *(TNF-alpha-induced protein 8), *IER3 *(immediate early response 3), *SERPINB2 *(serine or cysteine proteinase inhibitor B2), and *IRF2 *(interferon regulatory factor 2) (Table. 1d). This supports the fact that, in the initial stages of mast cell activation, several mediators produced are mainly for cell proliferation and survival [28]. Thus, FcεRI aggregation may enhance mast cell proliferation and survival, perhaps owing to the autocrine effects of the cytokines, growth factors, and antiapoptotic proteins, triggered by FcεRI aggregation.

### Cell adhesion and cytoskeleton remodeling

Another functional characteristic of immune-cell activation is the coordinated expression of genes involved in cell adhesion and cytoskeleton remodeling (Table. 1e). Of particular importance are the genes coding for proteins involved in cell motility, cytokinesis, endocytosis and exocytosis. We found at least 2-fold upregulation of various genes coding for proteins involved in cell adhesion, such as *FLRT2 *(fibronectin leucine rich transmembrane protein 2), *KAL1 *(Kallmann syndrome 1 sequence), *CD151 *(CD151 antigen), and *ALCAM *(activated leukocyte cell adhesion molecule); as well as for several gene-transcripts involved in cytoskeleton remodeling, including *RASAL1 *(RAS protein activator like 1), *ARHE *(RAS homolog gene family, member E), *ARF6 *(ADP-ribosylation factor 6), and *FLNB *(filamin B, β-actin binding protein 278) (Table. 1e). The expression of genes involved in cell adhesion and cytoskeleton remodeling is an essential step in immune-cell activation. Resting immune cells have cytoskeletal structures that sequester antigen, chemokine, and adhesion receptors in accessible regions of the plasma membrane. Upon activation, reorganization of the actin cytoskeleton leads to the formation of supramolecular activation clusters, bringing receptors and costimulatory molecules together, as well as important adaptor proteins that promote the sustained activation of the cell.

### Transcription factors and regulators of transcription

Stimulation of immune-effector cells through their antigen receptors initiates cell cycle entry and changes the gene expression pattern, a response generally referred to as "activation". We found that the genes for several transcription factors were upregulated during IgE-sensitization and FcεRI aggregation, including the transcription factors most active during an immune response, such as *NF*κ*B *and *NFAT *(Table. 1f). We observed an increase in the transcripts for the nuclear factor of kappa light polypeptide genes 1, alpha, and epsilon (NFκB 1, 1A, and 1E), and the nuclear factor of activated T cells, NFATC1 (Table. 1f). Other transcription factors upregulated were the oncogenes *MYC *(v-myc myelo-cytomatosis viral oncogene homolog), and *MAFF *(v-maf musculo-aponeurotic fibrosarcoma oncogene homolog F) (Table. 1f). Interestingly, the activities of NFκB and NFAT together are responsible for the transcription of many proinflammatory genes, including several genes coding for cytokines and chemokines [29,30].

### Signal transduction

During mast cell activation, many signaling molecules are engaged in diverse responses, ranging from calcium release from internal stores, degranulation, the generation of lipid-derived proinflammatory mediators and the production of cytokines and chemokines. In our study we observed that a substantial number of genes coding for intracellular signaling proteins were upregulated, by at least 2-fold (Table. 1g). These include genes for serine protein kinases of the Mitogen Activated Protein Kinase family, such as *MAP2K3 *and *MAP3K14*: members of this family of kinases not only play a role in mitogenesis, but can potentially lead to the activation of transcription factors. The gene coding for the tyrosine kinase *FYN *(FYN oncogene related to SRC, FGR, YES) was also upregulated (Table. 1g): FYN is a member of the Src-tyrosine kinase family; members of this family are critical for the signal transduction cascades triggered by all antigen receptors including by FcεRI, TCR, FcγRs, and the B-cell antigen receptor. Several genes coding for protein phosphatases were also upregulated, including several members of the dual specificity phosphatase family coding for DUSP1, DUSP2, and DUSP6 (Table. 1g). Moreover, we show here the upregulation of genes that code for oxidized low density lipoprotein receptor (*OLR1*), and for the low density lipoprotein receptor (*LDLR*) (Table. 1g), indicating a potential role for mast cells in cholesterol homeostasis. Of particular interest is the upregulation of the gene coding for the lipid kinase, sphingosine kinase 1 (*SPHK1*) (Table. 1g). We and others have previously reported that SPHK1 plays a critical role in the intracellular signaling pathways triggered by FcεRI in mast cells [28,31], and coordinates several physiological responses triggered by activated mast cells.

### Real time PCR

**Figure 4:**
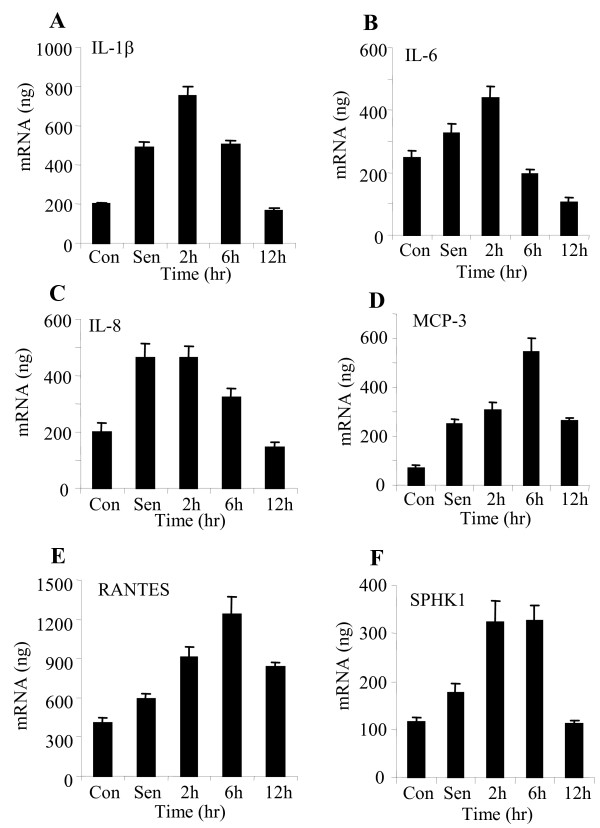
**Real-Time PCR of selected genes**. Comparison of gene expression from control/resting mast cells (Con); after mast cells were sensitized by human IgE (Sen), and following a time course of FcεRI aggregation for 2 hours (2 h), 6 hours (6 h) and 12 hours (12 h). 1 μg of total RNA was used from the same sample which has been used for microarray experiments at different time points for Light-Cycler Real-Time PCR. The concentrations of these transcripts were calculated using respective standard curves. (A) IL-1β expression; (B) IL-6 expression; (C) IL-8 expression; (D) MCP3 expression; (E) RANTES expression; and (F) SPHK1 expression. The data represent average ± the standard deviation of four different samples.

We confirmed our microarrays findings by Real time PCR on selected genes such as *IL-1*β *IL6*, *IL-8, MCP3, RANTES *and *SPHK1*, utilizing an aliquot of the same RNA sample that was used for the microarray experiments (Figure 4). The results showed that the messenger RNA for the selected genes follows a similar pattern of expression to that observed with the oligo-DNA microarray experiment, thus confirming the results and the quality of the data obtained with the high-density microarrays.

### Protein expression by ELISA

**Figure 5:**
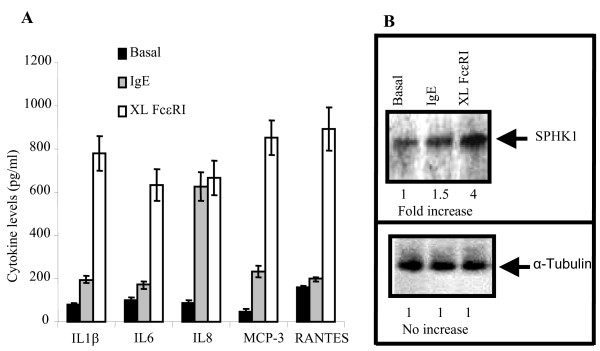
**Protein expression analysis**. **A. ELISA for selected Cytokines and Chemokines**. Cytokine and chemokine generation/release was determined from control-unstimulated mature mast cell (Basal); following IgE-sensitization (IgE) for 24 hours; and FcεRI aggregation by addition of the anti-human IgE to sensitized cells for 24 hours (XLFcεRI). Cell culture supernatants were analyzed for bioactive IL-1β; IL-6; IL-8; MCP-3 and RANTES by ELISA. Results shown are the mean plus the standard deviation of triplicate measurements and are representative of four separate experiments. **B. Western Blot analysis of SPHK1**. Upper panel; differential expression of SPHK1 was assessed by Western blot on lysates from control unstimulated cells (Basal); following IgE-sensitization (IgE) for 24 hours; and FcεRI aggregation by addition of the anti-human IgE to sensitized cells for 24 hours (XLFcεRI). Lower panel; the blots were probed for α-tubulin (control for equal loading). Results shown are representative of four separate experiments.

Mast cell activation also results in the sustained *de novo *production of pro-inflammatory cytokines and chemokines both of which may contribute to the inflammation and pathology underlying allergic disease as well as in innate and acquired immunity. The amounts of these cytokines were measured by ELISA and depicted in Figure 5A. FcεRI-triggered generation of IL-1β, IL-6, IL-8, CCL7 (MCP3) and CCL5 (RANTES), whereas IgE sensitization alone triggered smaller amounts of IL-1β and MCP3, high amount of IL-8, and very less amounts of IL-6 and RANTES (Figure 5A).

### Protein expression by Western blot

We verified the differential expression of SPHK1 by Western blot analysis (Figure 5B). Its levels of expression were found to be consistent with that of microarray as well as Real Time PCR results. Thus, these data together with Real Time PCR data validate microarray results.

### Mast cells show strong activation by FcεRI aggregation but not by IgE-sensitization

**Figure 6:**
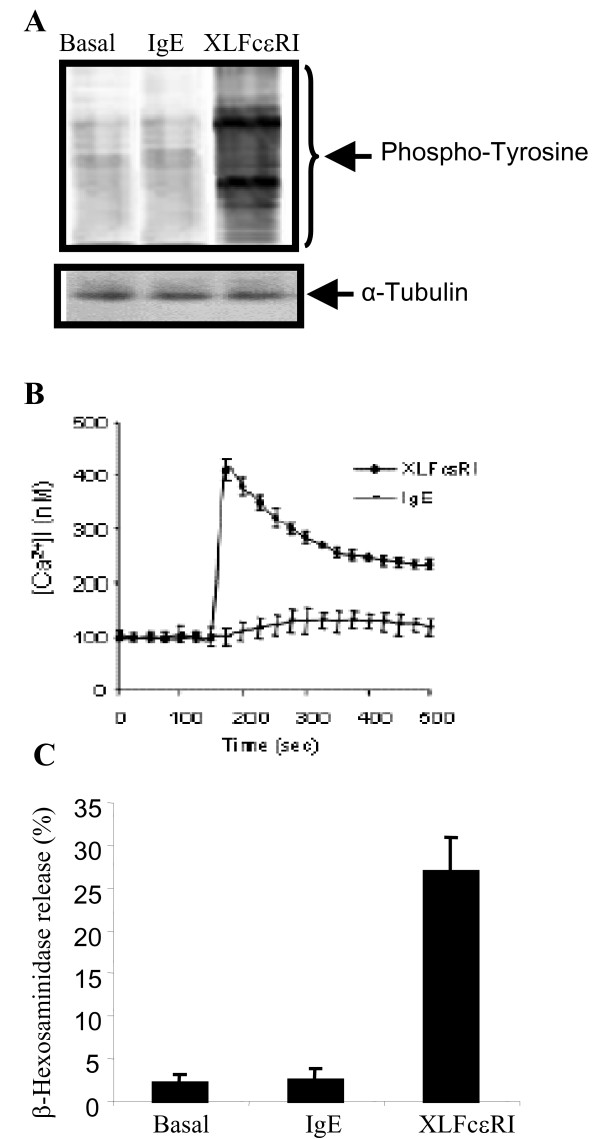
**Mast cell activation: tyrosine phosphorylation, calcium signals, degranulation**. **A. Analysis of overall protein phosphorylation on tyrosine residues**. Upper panel; overall tyrosine-phosphorylation pattern was analysed in cell extracts from: control unstimulated cells (Basal); cells treated with IgE alone for (IgE) for 5 min; and after FcεRI crosllinking for 5 min (XLFcεRI). Lower panel; the blots were probed for α-tubulin (control for equal loading). Results shown are representative of four separate experiments. **B. Levels of intracellular free calcium**. Intracellular calcium measurements of mast cells following addition of IgE alone (IgE); and following the addition of the anti-human IgE, to IgE-sensitized cells (XLFcεRI), the intracellular calcium levels were analyzed in a continuous reading for the timesstated in the graph. Results shown are the mean plus the standard deviation of triplicate measurements and are representative of four separate experiments. **C. Degranulation**. B-hexosaminidase release was determined from control-unstimulated mast cells (Basal); following monomeric-IgE-sensitization for 30 minutes (IgE); and following FcεRI aggregation by addition of the anti-human IgE to sensitized cells for 30 minutes (XLFcεRI). Results shown are the mean plus the standard deviation of triplicate measurements and are representative of four separate experiments.

Mast cell activation via FcεRI triggers exocytosis of granules containing pre-formed inflammatory mediators in a tyrosine kinase and calcium dependent manner. Here we studied, whether monomeric-IgE alone, may activate FcεRI intracellular signaling pathways, leading to physiological responses of mast cells, by analyzing the overall tyrosine phosphorylation; fluctuations in cytosolic Ca^2+ ^concentration; and degranulation by measuring β-hexosaminidase release (Fig. 6A,B &6C). We show here that in our experimental setting monomeric-IgE alone is not able to trigger any changes on the overall protein-tyrosine phosphorylation patterns compared with resting cells; nor was it able to trigger calcium release from internal stores; neither degranulation. On the other hand FcεRI-aggregation did indeed trigger all these responses (Fig. 6A,B &6C).

## Discussion

Binding of IgE to FcεRI enhances the cell surface expression of FcεRI, as a result, its ability to promote the stabilization/accumulation of FcεRI on the mast cell surface in the presence of continued basal levels of protein synthesis [32,33]. It is possible that most of the enhanced IgE dependent functions that are observed after antigen or anti-IgE-induced FcεRI aggregation, in cells that have been sensitized with IgE, are a consequence of the higher level of FcεRI expression. However, a controversial question remains as to whether monomeric IgE can also have more direct effects on mast cell functions. Many studies over the years have shown no evidence that the binding of monomeric IgE can induce detectable signaling or production of mediators by mast cells. However, some groups have reported that monomeric IgE can enhance mast cell survival and trigger cytokine production [24,28,33]. In contrast, a recent study by Matsuda *et al *[24], fail to find any ability of IgE to enhance mast cell survival on withdrawal of SCF. Interestingly, the study by Matsuda *et al*, also showed that IgE sensitization alone can induce the upregulation of cytokines and chemokines at the protein level, namely IL-8 and MCP1 [24]. In agreement to this, we show that IL-8 is induced by IgE alone at the mRNA as well as at the protein level (table 1, and Figures 4 and 5), in contrast we could not detect any significant increment on MCP-1 levels, we can speculate that this difference could be due to the different amounts IgE used (1 μg/mL *vs *2.5 μg/mL). However, we also show the upregulation of various chemokines, including the MCP-1-related protein MCP-3, which was also upregulated by IgE alone (table 1, and Figures 4 and 5). In our present study, we found that several genes related to proliferation were upregulated by IgE alone (Table 1); however, whether these genes, if fully transcribed, may be able to trigger mast cell proliferation in the absence of SCF is not known.

The observation that IgE alone can induce the upregulation of a substantial number of genes encoding for cytokines and chemokines, has profound implications in our understanding of the role of mast cell in inflammation. Cytokines and chemokines share many activities, including the ability to induce fever and shock syndrome in animal models [27]. Cytokine and chemokine production are universal components of a wide range of disease states, including immune-complex-mediated conditions such as nephritis [34], arthritis [35], and acute graft rejection [36]. These data suggest a potential role for mast cells in triggering, or at least contributing to, strong inflammatory responses.

In is also interesting to mention that, several genes encoding for transcription factors were upregulated by monomeric-IgE and FcεRI aggregation. Perhaps the most prominent of these transcription factors, is NFκB. NFκB represents a family of related proteins which dimerize to form transactivating complexes [35]. NFκB dimmers are sequestered in the cytoplasm by interaction with inhibitory proteins (the IκBs). Various stimuli activate kinase signaling cascades that result in the phosphorylation and degradation of IκB, thereby releasing NFκB to translocate to the nucleus, where it activates transcription of target genes. Many studies have emphasized the role of this transcription factor in regulating genes at critical points in immune-cell development and activation [37]. Many NFκB targets are antiapoptotic [38], which may explain the importance of the NFκB pathway in oncogenesis and resistance to chemotherapy [39,40]. During an immune-response several genes are triggered by the NFκB, these include genes coding for the various proinflammatory molecules, such as MIPs, IL-1β, IL-6, IL-8, TNFα, GROα and other cytokines, chemokines and cell adhesion molecules ICAM, VCAM and selectins [41].

As immune cells progress through development and respond to antigenic challenge, they trigger signal transduction pathways that alter their cellular functions and the activity of transcription factors, changing their effector functions and their gene expression profiles. During mast cell activation, many signaling molecules are engaged in diverse responses, ranging from calcium release from internal stores, degranulation, the generation of lipid-derived proinflammatory mediators and the production of cytokines and chemokines. In our study we observed that a substantial number of genes coding for intracellular signaling proteins were upregulated, by at least 2-fold, during mast cell stimulation (Table. 1g). Interestingly, we observed a substantial upregulation of the mRNA for sphingosine kinase 1 (*SPHK1*), even by IgE alone, this upregulation was also confirmed at the protein level. Sphingosine kinases are novel enzymes that phosphorylate sphingosine (a membrane lipid), to generate the bioactive molecule sphingosine-1-phosphate (SPP), which is implicated in several inflammatory responses. We and others have previously reported that SPHK1 plays a critical role in the intracellular signaling pathways triggered by FcεRI in mast cells [28,31], and coordinates several physiological responses triggered by activated mast cells. We showed that SPHK1 is involved in the calcium signals triggered by FcεRI aggregation in human mast cells, as well as playing a critical role for mast cell degranulation [31]. Previously, we reported a pivotal role for SPHK1 in monocyte activation by the high-affinity IgG receptor (FcγRI) [42,43], showing that SPHK is key in triggering calcium release from internal stores, and the activation of the phagocyte NADPH oxidase. Moreover, very recently we demonstrated the role of SPHK1 in inflammatory responses triggered by the anaphylatoxin C5a in human neutrophil and macrophages. These responses include: calcium signals, degranulation, cytokine production and chemotaxis triggered by C5a [44,45]. The observation that the gene encoding for SPHK1 is activated during IgE-sensitization of mast cells, coupled to the findings above may indicate a key role for SPHK1 in mast cell triggered responses.

The significance of this research supports the notion that, activation of mast cells appear to be linked to a wide range of pathologies, not only in allergies (as is widely recognized), but potentially in other inflammatory conditions. The method of global gene expression analysis using cDNA or oligo-DNA microarrays has proven to be a sensitive method to identify and define/redefine the molecular determinants of several human disorders, including cancer and autoimmune diseases, and has provided us with signatures of the immune response [41]. Using this technology, complemented with powerful analytical methods, we compared the gene expression profiles of human mast cells stimulated by IgE sensitization, and from a series of time points of FcεRI aggregation, with unstimulated/control human mast cells. Whether changes in gene expression, under these conditions, are representative of a pathological state is not currently known. It is also not known whether IgE/antigen and FcεRI aggregation will trigger the same set of genes in an organism, where a number of events may be activating mast cells at the same time. However, taken together, our data brings us better insights into the molecular basis of mast cell activation, and provides meaningful information, regarding the mechanisms by which mast cell activation may contribute to the overall activation of the immune response, having clinical implication for improving not only allergic conditions but potentially other inflammatory diseases, where mast cells may play a role.

## Conclusion

This study is an attempt to elucidate the molecular mechanisms which mast cells undergo during "priming" IgE sensitization and full activation by FcεRI aggregation in a global perspective. In conclusion, our present study provides information that mast cells, by generating a broad range of cytokines and chemokines, may be a potent contributor of the immune response by recruiting and/or activating other immune-effector cells including the activation of lymphocytes that may, in turn, continue the spreading of the inflammatory response. Moreover, changes in the gene expression pattern of transcription factors, intracellular signaling molecules, and cytoskeletal remodeling and anti-apoptosis pathways occur, which would also contribute to the amplification of the inflammatory response. Mast cells are well established innate immune-effector cells, and there is mounting evidence to, at least, suggest that mast cells may contribute to the development of acquired immunity [46,47], whether in host defense or in allergic or autoimmune diseases. It will be pivotal to define in more detail whether and under which circumstances mast cells may influence the development and/or magnitude of acquired immune responses.

## Methods

Unless specifically stated all materials and reagents were purchased from Sigma-Aldrich (Singapore).

### Cell culture

Human umbilical cord blood (CB) samples were collected from normal full-term deliveries of informed individuals with formal consents, meeting the Universality Institutional Review Board guidelines, for research using human samples. CD34^+ ^haematopoietic progenitor-cells were harvested using MACS cell isolation kit (Miltenyi Biotec), following the manufacturer's instructions.

The isolated CD34^+ ^haematopoietic progenitor-cells were cultured for 5-6-weeks in the presence of 100 ng/ml of Stem Cell Factor (SCF Cat: 300-07, PeproTec, Rocky Hill, NJ), and for the first week this was supplemented with 10 ng/ml of Interleukin-3 (IL-3 Cat: 200-03, PeproTec, Rocky Hill, NJ). Cells were shown to be differentiated by staining them for specific mast cell markers as follows: for mast cells chymase, with an anti-human chymase mAb (IgG1-MAB1254, Chemicon, Temecula, CA), and FITC-conjugated secondary antibody (anti-mouse IgG-FITC, Sigma-Aldrich, Singapore); for c-Kit with anti-human c-Kit mouse monoclonal PE-conjugated (Cat. No. 555714; clone YB5.B8, BD Biosciences – Pharmigen, Singapore), isotype control anti-mouse IgE-PE (Cat. No. 555749; clone MOPC-21, BD Biosciences – Pharmigen, Singapore); and for FcεRI cell-surface expression with anti-human FcεRI polyclonal (Rabbit-IgG-ab31494; Abcam, Cambridge, UK) and FITC-conjugated secondary antibody (anti-rabbit IgG-FITC, Sigma-Aldrich, Singapore) also used as isotype control, and analyzed immediately using a Coulter Epics-Elite ESP Flow Cytometer (Beckman, Germany). Purity was estimated at >97%. The differentiated mast cells were plated in 6 well plates and allowed to rest for 24 hr.

### Mast cell stimulation and RNA extraction

After differentiation, mast cells were plated in 6 well plates and allowed to rest for 24 hr. Cells in all wells, except the control well, were sensitized with human monomeric IgE (1 μg/ml, IgE, Cat: 30-AI05, Lot number A01071004, Fitzgerald, Concord, MA) overnight. FcεRI aggregation was carried out by incubating the cells with monoclonal mouse-anti-human IgE (1 μg/ml, Anti-human-IgE, Cat: MCA2115, clone 4C3, Serotec, Oxford, UK) at 37°C, for 2 hr, 6 hr and 12 hr. RNA was extracted from all the samples using the Qiagen RNeasy mini kit (Qiagen, Valencia, CA). Integrity of RNA was checked by formamide gel electrophoresis; quantification of RNA was carried out by measuring the A_260 nm_.

### Labeling and hybridization

Labeling and hybridization was carried out as previously described [46]. Briefly, 8 μg of total RNA from each sample was used to synthesize double stranded cDNA using T7-(dT24) oligonucleotide primer and Superscript Reverse transcriptase (Invitrogen). The resultant cDNA was purified and 1 μg of purified cDNA was labeled with biotin by transcription *in vitro*. The labeled cRNAs were, fragmented in the presence of metal ions and then hybridized to HG-Focus array (Affymetrix), following hybridization the gene chips were washed and stained after which the chips were scanned by Gene Array Scanner (Agilent technologies).

### Microarray data collection and analysis

Data collection and analysis was carried out using MicroArray Suite 5.0 (MAS) (Affymetrix). The absolute data (signal intensity, detection call and detection P-value) were exported into GeneSpring v7.0 (Silicon Genetics, Redwood City, CA, USA) software for analysis by parametric test based on cross gene error model (PCGEM). The ANOVA approach has been used to find differentially expressed genes (*P *< 0.05). The Benjamini and Hochberg False Discovery Rate multiple testing correction was applied.

### Gene expression profile clustering

Agglomerative average-linkage hierarchical clustering of the five different experimental were obtained for selected groups of genes with Gene Spring 7.0 software (Silicon Genetics, Redwood City, CA, USA) using standard correlation as similarity matrix.

### Real-time Quantitative PCR

Real-PCR was performed, as previously described [46], using 1 μg of total RNA from the same samples used for microarray experiments. PCR was performed for transcripts of IL-1β (Primers: forward 5'-ATG GCA GAA GTA CCT AAG CTC GC-3', reverse 5'-ACA CAA ATT GCA TGG TGA AGT CAG TT-3'); IL-6 (Primers: forward 5'-ATG AAC TCC TTC TCC ACA AGC GC-3', reverse 5' GAA GAG CCC TCA GGC TGG ACT G-3'); IL-8 (Primers: forward 5'-ATG ACT TCC AAG CTG GCC GTG GCT-3', reverse 5'TCT CAG CCC TCT TCA AAA ACT TCT C-3'); MCP-3 (Primers: forward 5'-TCC AAG GCT TTA TGT TCA AA-3', reverse 5'-ACT GAA CTG AAA ACA AGC CA-3'); RANTES (Primers: forward 5'-GCT GTC ATC CTC ATT GCT ACT G-3', reverse 5'-TCG AAC TCC TGA CCT CAA GTG ATC-3'); and for SPHK1 (Primers: forward 5'-TGA ACC CGC GCG GCA AGG GC-3', reverse 5'-GGT CAG CCG GCG CCA TCC ACG-3').

For amplicon detection, the Light Cycler RNA Master SYBR Green Kit (Roche) was used as described by the manufacturer. PCRs were performed in a LightCycler^® ^instrument (Roche) as follows: reverse transcription at 61°C for 20 min, initial denaturation at 95°C for 2 min; amplification for 45–65 cycles of denaturation (95°C, 5s, ramp rate 20°C/s), annealing (optimal temperature, 5s, ramp rate 20°C/s) and extension (72°C, product length [bp]/25 s, ramp rate 2°C/s). A single online fluorescence reading for each sample was taken at the end of extension step. Quantitative results were expressed by identification of the second derivative maximum points, which marked the cycles where the second derivatives of the fluorescence signal curves are at maximum. These points were expressed as fractional cycle numbers. Then, these cycle numbers were plotted against the logarithm of the concentrations of serially 2-fold diluted standard samples to obtain a standard curve. The concentrations of unknown samples were calculated by extrapolation from this standard curve. Positive sample specificity was confirmed by determining the melting curve (95°C, 5s, ramp rate 20°C/s; 68°C, 15s, ramp rate 20°C/s; 95°C, 0s, ramp rate 0.1°C/s, continuous measurement).

### Cytokine detection

Supernatants from control cells, cells sensitized, and cells following FcεRI aggregation, were collected and stored at -20C until use. IL-1β, IL-6, IL-8, MCP-3 and RANTES levels in the supernatants were evaluated using ELISA (R&D Systems Inc., MN, USA) following the manufacturer's instructions.

### Gel electrophoresis and Western blots

Western blots were carried out as previously done [31]. Briefly, 40 μg of lysate for each sample was resolved on 10% polyacrylamide gels (SDS-PAGE) under denaturing conditions and then transferred to 0.45 μm nitrocellulose membranes.

For overall tyrosine phosphorytaion, the blots were probed using a specific monoclonal anti-phosphotyrosine primary antibody (P-Tyr, Sc-7020, Santa Cruz, CA, USA), and an anti-mouse HRP-conjugated secondary antibody (anti-mouse IgG-HRP, A-4416, Sigma). Bands were visualized using the ECL Western Blotting Detection System (Amersham, Singapore).

For SPHK1 expression, the blots were probed using a rabbit polyclonal anti-SPHK1 primary antibody (anti-SPHK1, X1627P, Exalpha, MA, USA), and HRP-conjugated secondary antibody (anti-rabbit IgG-HRP, sc-2004, Santa Cruz, CA, USA). For loading control the blots were probed with a monoclonal anti α-tubulin (anti-α-tubulin, sc-5286, Santa Cruz, CA, USA), and an anti-mouse HRP-conjugated secondary antibody (anti-mouse IgG-HRP, A-4416, Sigma). Bands were visualized using the ECL Western Blotting Detection System (Amersham, Singapore), and quantified by densitometry analysis.

### Cytosolic Ca^2+^

Cytosolic calcium was measured as described previously [31]. Briefly, cells were loaded with 1 μg/ml Fura2-AM (Molecular Probes, Leiden, The Netherlands) in PBS, 1.5 mM Ca^2+ ^and 1 % BSA. After removal of excess reagents by dilution and centrifugation (in PBS), the cells were resuspended in PBS containing 1.5 mM Ca^2+ ^and 1 % BSA, for 30 min; or in PBS containing 1.5 mM Ca^2+^, 1 % BSA, and human-monomeric IgE (1 μg/ml) for sensitization, for 30 min. After removal of excess IgE by dilution and centrifugation (in PBS), the cells were resuspended in 1.5 mM Ca^2+ ^supplemented PBS and warmed to 37°C in the cuvette; Unsensitazed cells were placed in the cuvette and cytosolic calcium was measured before and after the addition of monomeric IgE. IgE-sensitized cells were placed in the cuvette and FcεRI was crosslinked by addition of mouse-anti-human IgE (1 μg/ml). Fluorescence was measured at 340 and 380 nm.

### β-hexosaminidase release

Degranulation was measured using as previously described [31]. Briefly, an aliquot of cells was resuspended in PBS containing 1.5 mM Ca^2+ ^and 1 % BSA, and incubated with monomeric IgE for 30 min at 37°C. Another aliquot of cells was resuspended in PBS containing human-monomeric IgE (1 μg/ml) for sensitization, 1.5 mM Ca^2+ ^and 1 % BSA, for 30 min. After removal of excess IgE by dilution and centrifugation (in PBS), the cells were resuspended in 1.5 mM Ca^2+ ^supplemented PBS, and FcεRI was crosslinked by addition of mouse-anti-human IgE (1 μg/ml) to cells for 30 min at 37°C.

Following the incubation, 50 μl of supernatant, was incubated with 200 μl of 1 mM *p-*nitrophenyl *N-*acetyl-β-D-glucosaminide for 1 hr at 37°C. The total β-hexosaminidase concentration was determined by a 1:1 extraction of the remaining buffer and cells with 1% Triton X-100; a 50 μl aliquot was removed and analyzed as described. Reactions were quenched by addition of 500 μl of 0.1 M sodium carbonate buffer. The enzyme concentration was determined by measuring the OD at 400 nm. β-hexosaminidase release was represented as a percent of total enzyme.

### Flow cytometry analysis for mast cell markers

To analyze the expression of the intracellular mast cell chymase, 1 × 10^6^cells were washed with ice cold PBS, fixed and permeabilised using the FIX and PERM reagents from Caltag (Caltag Laboratories, Burlingame, CA) as follows: after washing, samples were resuspended in 100 ul of Reagent A (Fixation medium) and incubated for 15 min at RT. The cells were then washed twice with ice-cold PBS, and resuspended in 100 ul of Reagent B (Permeabilization medium) and incubated for 15 min at RT. Cells were washed twice and resuspended in 100 μl of PBS/1% FBS and 5 μl of the anti-human chymase mAb (IgG1-MAB1254, Chemicon, Temecula, CA) was added and samples were incubated for 20 min at RT. Samples were washed twice in ice-cold PBS, then resuspended in PBS/1% FBS and 5 μl of FITC-conjugated secondary antibody (anti-mouse IgG-FITC, Sigma-Aldrich, Singapore) was added, and incubated in dark for an 30 min at RT. Samples were washed twice with ice-cold PBS and resuspended in 100 μl of PBS/1% FBS for immediate analysis.

To analyze the cell surface expression of cKit and FceRI, the samples were initially processed as above except that the permeabilisation step was omitted. For c-Kit the primary antibody was an anti-human c-Kit mouse monoclonal (MCA1841, clone 104D2, Serotec, Oxford, UK), and the secondary antibody was a FITC-conjugated (anti-mouse IgG-FITC, Sigma-Aldrich, Singapore). For FcεRI cell-surface expression, the cells were labeled with the primary anti-human FcεRI polyclonal (Rabbit-IgG-ab31494. Abcam, Cambridge, UK), and the secondary antibody was a FITC-conjugated (anti-rabbit IgG-FITC, Sigma-Aldrich, Singapore). All the samples were analyzed by flow cytometry was using a FACSCalibur machine (BD biosciences), and the data analysed using the Cell Quest™ Pro Software.

## Authors' contributions

MJ carried out data analysis and prepared the microarray-data table and figures. HKT isolated the progenitor cells, differentiated the Mast cells and carried out the receptor crosslinking. RR carried out the RNA extraction, labeling and microarray hybridization. LZ carried out RT-PCR. KKC and MR provided the cord blood. AJM designed the study and drafted the manuscript. All authors read and approved the final manuscript.

## Figures and Tables

**Table 1:** Differentiated, human cord blood-derived, mast cells were sensitized overnight by the addition of monomeric IgE followed by crosslinking the high affinity IgE receptors, FcεRI using anti IgE for 2 h, 6 h and 12 h. Gene expression profile at each time point were analyzed and 115 genes, which were upregulated 2 fold or more (over control/unstimulated cells) atleast in one point have been selected and classified according to their biological functions as a) Cytokines and cytokine receptors b) Chemokine and chemokine receptors c) Other immunoregulatory genes d)Cell proliferation and anti-apoptosis e)Adhesion and cytoskeleton remodeling f)Transcription factors and regulators g) Signal transduction h) Other genes. Fold change is expressed as fold-increase over control/unsensitized samples, and representative of four separate experiments.

**a. Cytokines and cytokine receptors**						
**Genbank ID**	**Gene symbol**	**Gene name**	**IgE Sens**	**2 h**	**6 h**	**12 h**

M15330	*IL1B*	Interleukin 1, beta	3.3	4.8	1.5	-2.0
NM_000600.1	*IL6*	Interleukin 6	2.0	2.6	1.9	-1.3
M37435	*CSF1*	Colony stimulating factor 1	1.6	4.5	1.9	-1.4
NM_000877.1	*IL1R1*	Interleukin 1 receptor, type I	1.4	2.2	1.9	-1.2
AI983115	*IL27RA*	Class I cytokine receptor	1.4	1.3	2.6	1.1
NM_001561.2	*TNFRSF9*	Tumor necrosis factor receptor superfamily, member 9	1.2	1.5	2.5	1.7
NM_016639.1	*TNFRSF12A*	Tumor necrosis factor receptor superfamily, member 12A	1.5	2.4	-1.3	-1.9
X52015	*IL1RN*	Interleukin 1 receptor antagonist	1.1	2.4	2.6	1.4

**b. Chemokine and chemokine receptors**						

**Genbank ID**	**Gene symbol**	**Gene name**	**IgE Sens**	**2 h**	**6 h**	**12 h**

NM_002984.1	*CCL4*	Chemokine (C-C motif) ligand 4	3.5	4.7	1.6	-3.0
NM_001511.1	*CXCL1*	Chemokine (C-X-C motif) ligand 1	2.3	2.5	1.1	-2.9
NM_006273.2	*CCL7*	Chemokine (C-C motif) ligand 7	2.1	3.6	5.3	2.2
NM_002090.1	*CXCL3*	Chemokine (C-X-C motif) ligand 3	1.6	2.5	1.6	-1.8
NM_002985.1	*CCL5*	Chemokine (C-C motif) ligand 5	1.6	1.8	2.1	1.8
AF015524	*CCRL2*	Chemokine (C-C motif) receptor-like 2	1.5	2.0	2.0	1.2
AF043337	*IL8*	Interleukin 8	2.1	2.1	1.5	-1.5

**c. Other immunoregulatory genes**						

**Genbank ID**	**Gene symbol**	**Gene name**	**IgE Sens**	**2 h**	**6 h**	**12 h**

S59049	*BL34*	B cell activation gene	3.4	6.8	-2.3	-3.9
NM_002309.2	*LIF*	Leukemia inhibitory factor (Cholinergic differentiation factor)	-1.3	5.1	-1.3	-4.0
L07555	*CD69*	CD69 antigen	-1.1	4.1	-1.0	-1.7
AF036906	*LAT*	Linker for activation of T cells	1.9	2.3	2.3	1.7
NM_004233.1	*CD83*	CD83 antigen	2.4	3.0	1.6	-1.2
NM_000675.2	*ADORA2A*	Adenosine A2a receptor	2.1	3.8	1.4	-1.3
NM_001311.1	*CRIP1*	Cysteine-rich protein 1	1.5	2.6	2.7	1.3
M16276	*HLA-DQB1*	Major histocompatibility complex, class II, DQ beta 1	1.9	2.0	1.7	1.2
AW007185	*HKE2*	HLA class II region expressed gene KE2	1.4	1.4	2.2	1.9
NM_005658.1	*TRAF1*	TNF receptor-associated factor 1	1.5	2.5	1.8	-1.0
NM_005601	*NKG7*	Natural killer cell group 7 sequence	1.4	2.2	1.3	1.3
NM_002852.1	*PTX3*	Pentaxin-related gene, rapidly induced by IL-1 beta	1.8	2.5	1.5	-2.1
M31933	*FCGR2B*	Fc fragment of IgG, low affinity IIb, receptor for (CD32)	2.0	2.0	1.3	-1.1
NM_007115.1	*TNFAIP6*	Tumor necrosis factor, alpha-induced protein 6	2.0	2.4	2.1	-1.1
NM_000956.1	*PTGER2*	Prostaglandin E receptor 2 (subtype EP2)	2.0	2.2	1.3	-1.5
NM_013252.1	*CLECSF5*	C-type lectin, superfamily member 5	1.6	2.3	2.5	1.2
NM_004951.1	*EBI2*	Epstein-Barr virus induced gene 2 (lymphocyte-specific GPCR)	1.4	2.0	-1.7	-2.0
NM_018643.1	*TREM1*	Triggering receptor expressed on myeloid cells 1	1.4	2.2	2.5	1.7
U37546	*BIRC3*	Baculoviral IAP repeat-containing 3	1.4	2.0	1.1	-1.1
NM_002000.1	*FCAR*	Fc fragment of IgA, receptor for	1.4	2.0	1.7	-1.9
NM_003264.1	*TLR2*	Toll-like receptor 2	1.4	2.0	2.0	-1.6
NM_002114.1	*HIVEP1*	Human immunodeficiency virus type I enhancer binding protein 1	2.0	1.6	-1.2	-1.6

**d. Cell proliferation and anti-apoptosis**						

**Genbank ID**	**Gene symbol**	**Gene name**	**IgE Sens**	**2 h**	**6 h**	**12 h**

NM_006732.1	*FOSB*	FBJ murine osteosarcoma viral oncogene homolog B	2.1	3.9	-3.0	-4.9
NM_004430.1	*EGR3*	Early growth response 3	1.8	7.5	-1.4	-3.3
AF001294	*PHLDA2*	Tumor suppressing subtransferable candidate 3	2.6	3.8	1.7	-1.2
NM_002575.1	*SERPINB2*	Serine (or cysteine) proteinase inhibitor, clade B (ovalbumin), member 2	1.9	3.8	5.2	-1.1
M60485	*FGFR1*	Ffibroblast growth factor receptor 1 (fms-related tyrosine kinase 2, Pfeiffer syndrome)	2.3	2.2	2.7	1.5
NM_000399.2	*EGR2*	Early growth response 2 (Krox-20 homolog, Drosophila)	1.6	2.7	1.6	-1.9
NM_005542.3	*INSIG1*	Insulin induced gene 1	1.7	2.8	1.6	-1.2
NM_002607.1	*PDGFA*	Platelet-derived growth factor alpha polypeptide	1.8	2.4	1.7	1.1
NM_003897.1	*IER3*	Immediate early response 3	1.8	3.0	-1.2	-2.3
NM_002608.1	*PDGFB*	Platelet-derived growth factor beta polypeptide	1.2	2.0	-1.2	1.0
NM_006763.1	*BTG2*	BTG family, member 2	1.5	2.0	-1.1	-1.9
NM_005655.1	*TIEG*	TGFB inducible early growth response	1.8	1.9	-1.8	-2.4
NM_004073.1	*CNK*	Cytokine-inducible kinase	1.2	1.8	-1.5	-2.4
BF575514	*PBEF1*	Pre-B-cell colony-enhancing factor	1.6	1.9	-1.3	-3.2
NM_006290.1	*TNFAIP3*	TNF-induced protein 3	2.7	3.4	1.8	-1.0
BC005352	*TNFAIP8*	TNF-induced protein 8	2.0	1.6	1.3	1.3
NM_023009.1	*MLP*	MARCKS-like protein	1.5	1.7	2.5	1.1
NM_001878.2	*CRABP2*	Cellular retinoic acid binding protein 2	1.1	1.2	1.4	2.0
NM_002199.2	*IRF2*	Interferon regulatory factor 2	-1.5	-1.7	1.2	2.0

**e. Adhesion and cytoskeleton remodeling**						

**Genbank ID**	**Gene symbol**	**Gene name**	**IgE Sens**	**2 h**	**6 h**	**12 h**

NM_004658.1	*RASAL1*	RAS protein activator like 1 (GAP1 like)	2.2	2.2	3.6	2.3
NM_013231.1	*FLRT2*	Fibronectin leucine rich transmembrane protein 2	1.4	2.3	2.3	2.4
NM_003078.1	*SMARCD3*	SWI/SNF related, matrix associated, actin dependent regulator of chromatin, subfamily d, member 3	2.2	1.9	1.2	1.1
BG054844	*ARHE*	Ras homolog gene family, member E	1.3	2.2	1.9	-1.0
NM_000216.1	*KAL1*	Kallmann syndrome 1 sequence	1.4	1.4	1.3	2.0
M62994	*FLNB*	Filamin B, beta (actin binding protein 278)	1.2	1.4	2.0	1.0
NM_004357.1	*CD151*	CD151 antigen	1.0	1.2	2.1	-1.1
AA243143	*ARF6*	ADP-ribosylation factor 6	2.1	1.3	1.1	1.0
NM_001627.2	*ALCAM*	Activated leukocyte cell adhesion molecule	1.1	1.1	1.7	2.0

**f. Transcription factors and regulators**						

**Genbank ID**	**Gene symbol**	**Gene name**	**IgE Sens**	**2 h**	**6 h**	**12 h**

AL021977	*MAFF*	V-maf musculoaponeurotic fibrosarcoma oncogene homolog F (avian)	2.6	6.3	1.2	-2.2
AF060154	*MSC*	Musculin (activated B-cell factor-1)	1.8	2.4	1.9	1.1
M55643	*NFKB1*	Nuclear factor of kappa light polypeptide gene enhancer in B-cells 1 (p105)	1.7	2.1	1.5	-1.0
NM_001706.1	*BCL6*	B-cell CLL/lymphoma 6	2.4	2.0	1.5	1.2
U08015	*NFATC1*	Nuclear factor of activated T-cells, cytoplasmic, calcineurin-dependent 1	1.3	2.3	-1.1	-1.1
NM_001674.2	*ATF3*	Activating transcription factor 3	1.3	2.0	-1.0	-1.6
AI078167	*NFKBIA*	Nuclear factor of kappa light polypeptide gene enhancer in B-cells inhibitor, alpha	1.9	2.0	1.3	-1.5
NM_004556.1	*NFKBIE*	Nuclear factor of kappa light polypeptide gene enhancer in B-cells inhibitor, epsilon	1.7	2.0	1.1	-1.2
NM_002467.1	*MYC*	V-myc myelocytomatosis viral oncogene homolog (avian)	1.7	2.1	1.1	-1.3
NM_001423.1	*EMP1*	Epithelial membrane protein 1	1.4	1.6	2.1	1.3
NM_012081	*ELL2*	ELL-related RNA polymerase II, elongation factor	1.7	1.3	2.6	1.3
NM_001067.2	*TOP2A*	Topoisomerase (DNA) II alpha 170kDa	2.0	1.0	1.2	1.3
AF078847	*GTF2H2*	General transcription factor IIH, polypeptide 2, 44kDa	-1.0	1.1	2.0	-1.1

**g. Signal transduction**						

**Genbank ID**	**Gene symbol**	**Gene name**	**IgE Sens**	**2 h**	**6 h**	**12 h**

NM_004418.2	*DUSP2*	Dual specificity phosphatase 2	4.3	7.2	1.8	1.5
NM_000361.1	*THBD*	Thrombomodulin	2.5	4.5	1.0	-1.7
S77154	*NR4A2*	Nuclear receptor subfamily 4, group A, member 2	1.3	4.1	-1.0	-2.1
NM_005261.1	*GEM*	GTP binding protein overexpressed in skeletal muscle	1.2	2.7	1.1	-1.1
AF035776	*OLR1*	Oxidised low density lipoprotein (lectin-like) receptor 1	2.0	2.6	3.1	-1.0
NM_000161.1	*GCH1*	GTP cyclohydrolase 1 (dopa-responsive dystonia)	2.0	2.4	1.7	-1.9
NM_021972.1	*SPHK1*	Sphingosine kinase 1	1.4	2.1	2.7	-1.0
NM_005001.1	*NDUFA7*	NADH dehydrogenase (ubiquinone) 1 alpha subcomplex, 7, 14.5kDa	1.6	1.6	2.0	1.4
M60278	*HBEGF*	Heparin-binding EGF-like growth factor	1.1	2.4	-2.1	-5.0
U77914	*JAG1*	Jagged 1 (Alagille syndrome)	1.4	2.5	1.8	1.0
NM_000527.2	*LDLR*	Low density lipoprotein receptor	1.6	2.0	-1.8	-1.4
NM_005904.1	*MADH7*	MAD, mothers against decapentaplegic homolog 7 (Drosophila)	1.7	1.9	-1.2	-2.0
AF070528	*MALT1*	Mucosa associated lymphoid tissue lymphoma translocation gene 1	1.5	2.1	1.2	-1.2
NM_005842.1	*SPRY2*	Sprouty homolog 2 (Drosophila)	1.1	2.2	1.4	-1.3
BC003143	*DUSP6*	Dual specificity phosphatase 6	1.2	2.2	1.2	-1.4
AA780381	*MAP2K3*	Mitogen-activated protein kinase kinase 3	-1.0	2.1	-1.0	-1.7
NM_001881.1	*CREM*	cAMP responsive element modulator	1.2	2.0	-1.0	-1.1
NM_004417.2	*DUSP1*	Dual specificity phosphatase 1	1.6	1.8	-2.3	-3.3
NM_003954.1	MAP3K14	Mitogen-activated protein kinase kinase kinase 14	1.5	2.0	1.3	1.4
AF305083	*FUT4*	Fucosyltransferase 4 (alpha (1,3) fucosyltransferase, myeloid-specific)	2.0	1.6	1.2	-1.5
BE327172	*JUN*	V-jun sarcoma virus 17 oncogene homolog	1.7	1.5	1.4	2.0
L06633	*PSCDBP*	Pleckstrin homology, Sec7 and coiled/coil domains, binding protein	1.3	1.6	2.0	1.1
M14333	*FYN*	FYN oncogene related to SRC, FGR, YES	1.3	1.5	3.7	1.6
NM_005023.1	*PGGT1B*	Protein geranylgeranyltransferase type I, beta subunit	1.7	1.4	1.5	2.1
NM_006296.1	*VRK2*	Vaccinia related kinase 2	1.3	1.4	2.0	-1.1
AF127481	*proto-LBC*	A kinase (PRKA) anchor protein 13	1.4	-1.1	1.3	2.0
NM_003318.1	*TTK*	TTK protein kinase	1.6	-1.1	2.1	1.6

**h. Other genes**						

**Genbank ID**	**Gene symbol**	**Gene name**	**IgE Sens**	**2 h**	**6 h**	**12 h**

NM_003633.1	*ENC1*	Ectodermal-neural cortex (with BTB-like domain)	2.2	3.1	1.1	1.4
NM_004694.1	*SLC16A6*	Solute carrier family 16 (monocarboxylic acid transporters), member 6	2.6	2.5	1.0	1.4
AA451996	*HIST2H2AA*	H2A histone family, member O	2.3	2.4	2.4	1.4
AF182273	*CYP3A4*	Cytochrome P450, subfamily IIIA (niphedipine oxidase), polypeptide 4	1.2	2.0	1.0	1.1
BC002649	*HIST1H1C*	Histone 1, Hic	2.1	1.8	2.0	1.7
NM_006101.1	*HEC*	Highly expressed in cancer, rich in leucine heptad repeats	2.3	1.3	2.1	1.6
NM_004760.1	*STK17A*	Serine/threonine kinase 17a	1.4	1.8	2.0	-1.1
NM_020651.2	*PELI1*	Pellino homolog 1 (Drosophila)	2.2	1.4	1.4	1.0
L39833	*KCNAB1*	Potassium voltage-gated channel, shaker-related subfamily, beta member 1	2.4	1.4	1.1	1.4
BF223021	*B4GALT4*	UDP-Gal:betaGlcNAc beta 1,4-galactosyltransferase, polypeptide 4	2.0	1.2	1.5	1.2
